# Evaluation of Pediatric Surgical Site Infections Associated with Colorectal Surgeries at an Academic Children’s Hospital

**DOI:** 10.3390/healthcare8020091

**Published:** 2020-04-09

**Authors:** Kimberly Pough, Rima Bhakta, Holly Maples, Michele Honeycutt, Vini Vijayan

**Affiliations:** 1 Department of Pharmacy Practice, Arkansas Children’s Hospital, Little Rock, AR 72202, USA; MaplesHolly@uams.edu; 2 Department of Pharmacy Practice, St. Christopher’s Hospital for Children, Philadelphia, PA 19134, USA; 3 College of Pharmacy, University of Arkansas for Medical Sciences, Little Rock, AR 72205, USA; RBhakta@uams.edu; 4 Department of Infection Prevention and Hospital Epidemiology, Arkansas Children’s Hospital, Little Rock, AR 72202, USA; HoneycuttMD@archildrens.org; 5 Division of Infectious Diseases, Department of Pediatrics, Valley Children’s Hospital, Madera, CA 93636, USA; VVijayan@valleychildrens.org

**Keywords:** colorectal surgery, pediatric, surgical prophylaxis, antibiotic prophylaxis, surgical site infections

## Abstract

Appropriate use of antibiotic prophylaxis (AP) is a key measure for the prevention of surgical site infections (SSI) in colorectal surgeries; however, despite the presence of national and international guidelines, compliance with AP recommendations remains low. The purpose of this study is to evaluate compliance with recommendations for the use of AP in children undergoing colorectal surgeries and to evaluate the effectiveness of antibiotics in the prevention of SSI. We collected demographic and clinical characteristics of patients who underwent colorectal surgeries, as well as microbiological and antimicrobial susceptibility data for patients who developed SSI. AP data were collected and compared with national guidelines. Antibiotic dosing and duration were most frequently in concordance with national guidelines, while antibiotic timing and selection had the lowest rates of compliance. Twelve of the 192 colorectal procedures evaluated resulted in SSI. Only 2 of the 12 children with SSI received appropriate AP for all four categories evaluated. Eight cases that resulted in SSI were due to organisms not covered by the recommended AP. We identified multiple areas for the improvement of AP in children undergoing colorectal surgery. A multidisciplinary approach to development of standardized protocols, educational interventions, and EHR-based algorithms may facilitate or improve appropriate AP use.

## 1. Introduction

According to the Centers for Disease Control and Prevention (CDC), surgical site infections (SSI) are infections that occur at or near the surgical incision within 30 days of a procedure, or 90 days for specified procedures [1]. These infections occur in approximately 2–5% of patients undergoing inpatient surgery in the United States, and account for approximately 20% of healthcare-associated infections in adults as well as children [2]. SSI are associated with high morbidity and mortality rates, and increased durations of hospitalization and healthcare costs [2,3,4].

Colorectal surgeries are associated with a higher rate of SSI than for other kinds of surgeries, ranging from 5% to 45% due to exposure to the increased bacterial load in the colon and the rectum [3,4,5,6,7]. Current guidelines published by the CDC and the Healthcare Infection Control Practices Advisory Committee for the Prevention of Surgical Site Infection recommend appropriate utilization of systemic antibiotic prophylaxis (AP) within a surgical bundle as a key measure to prevent SSI among patients undergoing colorectal surgeries [8]. Appropriate AP in colorectal procedures is based mainly on 4 principles: (1) correct antibiotic selection; (2) correct dose; (3) timing of administration, including appropriate re-dosing for extended procedures; and (4) discontinuation of antibiotics when the procedure is completed and surgical site is closed, or no more than 24 h post-operatively. The effectiveness of AP in the prevention of SSI is well established. In 2016, the World Health Organization published evidence-based recommendations regarding the use of AP in the prevention of SSI [1,2,9,10,11,12]. However, despite the presence of international and national guidelines, compliance with AP for surgical procedures has been staggeringly low among patients undergoing colorectal procedures [13,14]. 

Clinical evidence in support of AP for the reduction of infectious complications following colorectal surgery is derived almost exclusively from adult literature. There are no well-controlled studies evaluating the efficacy of AP and compliance with surgical AP in children undergoing colorectal procedures. However, as children and adults have similar fecal bacterial concentration and microbiological profiles, there is little reason to suspect that current guidelines would not be adequate for children [3,15,16].

The purpose of this study is to evaluate the compliance of surgeons to national recommendations for use of AP in children undergoing colorectal surgeries with particular regard to antibiotic selection, dose, timing prior to incision and intraoperative re-dosing, and duration of postoperative antibiotic use and is to evaluate the effectiveness of antibiotics in the prevention of SSI in children undergoing colorectal surgical procedures. 

## 2. Materials and Methods 

### 2.1. Study Design and Setting

We performed a retrospective cohort study at Arkansas Children’s Hospital (ACH) in Little Rock, Arkansas. ACH is a 336-bed academic teaching hospital and serves as the largest children’s hospital in Arkansas. This project was conducted in accordance with the Declaration of Helsinki, and the institutional review board of the University of Arkansas for Medical Sciences approved this study on November 21, 2017 (Protocol number 207,026), using expedited review procedures. 

### 2.2. Study Population

The study population included all pediatric patients <18 years of age who underwent a colorectal procedure at ACH from 1 January 2015 to 31 December 2016. We excluded surgeries that were performed as the direct result of trauma and those surgeries without anesthesia records available. A list of potentially eligible patients was provided by the hospital infection prevention team. Patients were identified through chart review of colorectal procedures and application of the standardized National Healthcare Safety Network (NHSN) definitions for SSI at the time of reporting. SSI were defined and reported to NHSN for each procedure, including all SSI types: superficial, deep, and organ space. The wound class system used in NHSN is adapted from the American College of Surgeons wound classification schema and includes Clean, Clean-Contaminated, Contaminated, and Dirty/Infected [17]. SSI were determined through prospective surveillance by four infection control practitioners who are certified in infection prevention with 4–26 years of experience. This known subset of children provided the opportunity for assessment of antibiotic prophylaxis utilization.

### 2.3. Data Collection/Study Procedures

We performed a comprehensive review of medical records by using a standardized data collection instrument to identify demographic information and clinical characteristics of patients who underwent colorectal surgeries at ACH. Perioperative antibiotic use, dose, timing of first administration, and duration of prophylaxis were collected and compared with the American Society of Health-System Pharmacists (ASHP) guidelines for appropriate use of antibiotics for surgical prophylaxis [9]. Microbiological and antimicrobial susceptibility data for patients who developed an SSI post-operatively were obtained from our institution’s microbiology laboratory for the 2-year time period.

### 2.4. Definitions

Based on the ASHP guidelines, appropriate antibiotic selection for colorectal procedures was defined as those using one of the following regimens: (1) cefazolin and metronidazole, (2) ceftriaxone and metronidazole, (3) cefoxitin, (4) cefotetan, (5) ampicillin-sulbactam, or (6) ertapenem [9]. Alternative regimens for patients with a beta-lactam allergy included clindamycin or metronidazole with an aminoglycoside, aztreonam, or a fluoroquinolone [9]. Vancomycin could be used in the place of clindamycin for patients with a beta-lactam allergy [9]. Inappropriate antibiotic selection was defined as any other regimen administered preoperatively for the purposes of AP. Antibiotic dose was considered appropriate if the administered dose was within 10% of the guideline recommended dose.

Antibiotic timing was categorized as appropriate or inappropriate. Appropriate timing was defined as administration of the first dose of antibiotics within 60 min prior to surgical incision. However, given the pharmacokinetics of fluoroquinolones and vancomycin, timing of 120 min prior to incision was deemed appropriate for those antibiotics. Antibiotics not administered during these time periods were considered as inappropriate timing.

Re-dosing interval was assessed from the time of administration of the preoperative dose of the antibiotic and deemed appropriate when given within two half-lives of the agent administered, and deemed inappropriate when not administered or if there was a delay in administration. Continuation of AP for >24 h after surgery without an infectious indication was deemed as inappropriate duration.

### 2.5. Statistical Analysis

We performed descriptive analyses of the above variables by using SPSS version 24. Testing of proportions was performed by using a χ2 or Fisher exact test as appropriate. All reported *p* values are 2-tailed and were considered significant if *p* < 0.05.

## 3. Results

We evaluated 208 colorectal surgical procedures, of which 192 children met the inclusion criteria. Sixteen patients were excluded due to lack of anesthesia records. Of the 192 surgeries performed, 12 (6%) met the NHSN criteria for a surgical site infection; the overall SSI rate was 6.25 per 100 surgical procedures.

The median age of all patients was 4.9 months (range, 0–17.7 years), and 113 (59%) were male. Fifteen (8%) children were overweight or obese. One hundred seventy-five (91%) surgeries were categorized as scheduled or elective, and 17 (9%) were urgent or emergent. The median duration of surgery was 92 min (range, 20–579 min). The median duration of hospitalization was 13 days (range, 1–511 days). Of the 192 patients, 62 (32%) were hospitalized at least once in the previous year.

The types of surgeries most frequently performed included colorectal resection (44%), ostomy formation/revision (35%), ostomy closure (34%), exploratory laparotomy (28%), and small bowel resection (17%); most patients required multiple surgery types during their procedures. Table 1 shows the demographic, clinical, and surgical characteristics of the patients.

### 3.1. Assessment of AP Compliance

**Figure 1:**
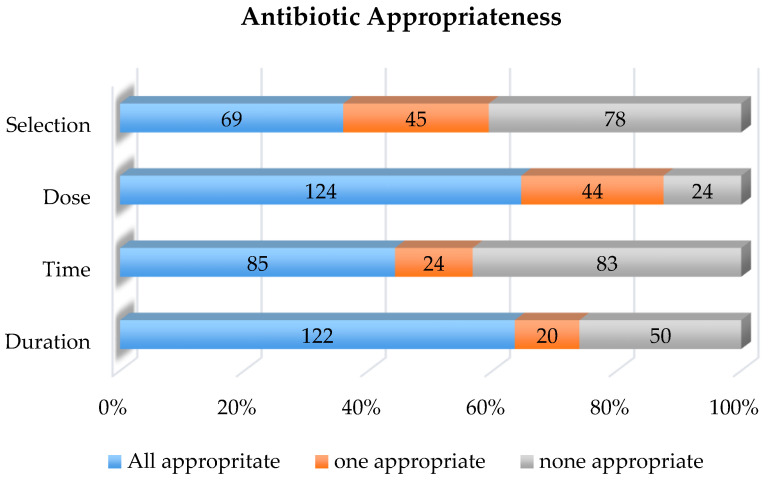
Appropriateness of antibiotic prophylaxis for children undergoing colorectal surgery as compared to national guideline recommendations.

The results of compliance with antimicrobial prophylaxis are shown in Figure 1. Appropriate antibiotic dosing and duration had the highest incidence of compliance at 65% and 64% of cases, respectively. Antibiotic timing and selection had the highest rates of non-compliance at 56% and 64% of encounters being non-compliant, respectively.

Antibiotic selection was found to be in concordance with both local and national recommendations in 36% of the cases (69/192). Combination of cefazolin and metronidazole was the most common appropriately used antibiotic regimen, accounting for 26% of all surgical cases. The most common inappropriate antibiotic regimens selected included cefazolin monotherapy and a combination of vancomycin with piperacillin-tazobactam. Vancomycin alone was administered to three patients, and metronidazole alone was administered to one patient. Anaerobic coverage was not included in the antibiotic regimen in 62% of patients. Thirty-five percent of patients for whom AP was not selected appropriately were on scheduled antibiotics for an infection prior to surgery, and hence, AP was perceived to be not indicated per surgical documentation. One patient did not receive any AP, and 6% of children that did not receive appropriate AP had a documented beta-lactam allergy.

With regard to antibiotic timing, 56% (107/192) of patients received AP outside of the recommended administration time. Of the 107 inappropriately timed antibiotics, 24 (22%) were due to vancomycin administration beyond the optimal time window prior to incision (range, 97–1144 min); 9 (8%) were due to emergency procedures. We found that 24 (22%) cases of inappropriate antibiotic timing were due to delay in the administration of metronidazole following the administration of cefazolin, ceftriaxone, or a fluoroquinolone. Of the 50 patients who were already on scheduled antibiotics prior to surgery, one received antibiotics at the appropriate time prior to incision, and three were appropriately re-dosed intraoperatively.

We found that dosing of AP was inappropriate in 68/192 (35%) of our patients. Dosing errors were noted most frequently for metronidazole; 71 patients in our cohort received metronidazole preoperatively, of which 29 (41%) received a higher dose than recommended, while 13 (18%) patients received a suboptimal dose of metronidazole. Of the 23 patients that required re-dosing of antibiotics, only 8 (35%) were re-dosed appropriately. The median surgical duration for procedures that required re-dosing was 177 min (range, 52–577 min).

AP duration was inappropriate in 70/192 (36%) cases. The duration of antibiotics after surgical procedure in patients whose post-operative prophylaxis was inappropriately prolonged was a median of 48.63 h (range, 31.33–182.62 h).

Overall, noncompliance with all four elements of antimicrobial prophylaxis was 44% among the 192 cases (Table 2).

### 3.2. Surgical Site Infections

Twelve children (6%) in our cohort developed SSI following colorectal surgery. Of these, 5 were superficial incisional, 2 were deep incisional, and 5 were organ/space infections. The percentages of clean-contaminated, contaminated, and dirty wounds in patients who developed infection were 25%, 17%, and 58%, respectively. Of the surgical cases resulting in SSI, 42% were emergent cases. Seventeen percent of infections occurred in patients who were obese and 25% occurred in patients who were premature. The median duration of surgery in cases resulting in SSI was 112.5 min (range, 76–206 min). Cases involving bowel resections accounted for 83% of all SSI.

Of the 12 patients with SSI, only two children received the correct AP for all four categories evaluated including selection, time, dose, and duration. Antibiotics were inappropriately selected in 4/12 (33%) children who developed an SSI. AP timing, duration, and dosing were inappropriate in 6/12 (50%), 5/12 (42%), and 2/12 (17%) cases, respectively.

**Figure 2:**
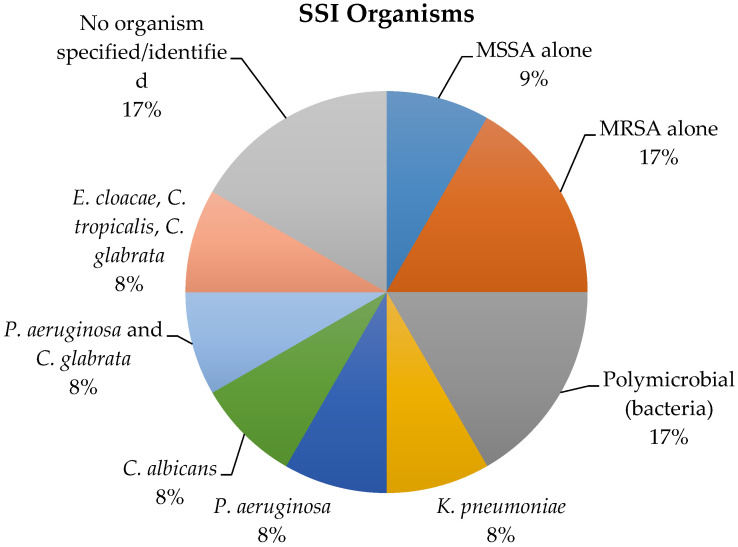
Organisms isolated in 12 children with a surgical site infection following colorectal surgery.

The organisms isolated in patients with SSI were methicillin-susceptible *Staphylococcus aureus* (MSSA), methicillin-resistant *Staphylococcus aureus* (MRSA), *Escherichia coli, Enterococcus faecalis, Pseudomonas aeruginosa, Klebsiella pneumoniae, Enterobacter cloacae, Candida albicans, Candida tropicalis*, and *Candida glabrata* (Figure 2).

Of the 10 children with an SSI wherein an organism was identified, 8 (80%) were not covered by the recommended AP. Of these 8 cases, 3 (38%) were due to *Candida* sp., and 5 (63%) were due to organisms that were resistant to the standard AP.

## 4. Discussion

We found lack of compliance with national guidelines in all four facets of AP in children undergoing colorectal procedures at our institution. Appropriate antibiotic selection and timing had the highest incidence of non-compliance, but we also identified non-compliance with antibiotic dosing and duration.

Antibiotic selection had the highest rate of non-compliance in our study with the correct antibiotic being chosen in only 36% of children undergoing colorectal surgeries. At our institution, the choice of AP is at the discretion of the surgeon or anesthesiologist. Lack of familiarity with the national guidelines for AP may be a barrier to appropriate antibiotic selection. In a study published by Friedman et al., excessively broad-spectrum antibiotics were chosen for clean operations [18]. This finding was similar to that seen in our study wherein concern for serious or severe infection prompted surgeons to unnecessarily choose broader spectrum antibiotics, thereby placing patients at risk for antibiotic resistance and fungal infections. The use of clinical decision support pathways and order sets that are incorporated into the electronic medical record may help guide antibiotic selection and prevent antibiotic overuse [14,19]. These order sets should be developed with the input of pharmacists and include optimal dosing for the chosen antibiotic, thereby potentially overcoming the AP dosing issues noted in our study. A multi-disciplinary approach including pharmacy, surgeons, nursing, and anesthesia for the development of the clinical decision support pathway may help to shed light on different perspectives of patient care and hold all members of the patient care team accountable for ensuring appropriate use of AP [14,19,20].

Nearly 60% of inappropriate AP in our study was due to incorrect timing. The ASHP guidelines recommend to administer antibiotics within one hour prior to surgical incision, or within 120 min for specific antibiotics [9]. We noted that when dual antibiotics were selected for AP, the second antibiotic, most often metronidazole, was either delayed or administered at or after the time of incision. The reason for this is unclear, but lack of familiarity with the pharmacokinetics of antibiotics may be a contributing factor. Tan et al. reported that AP was perceived as a low priority when compared to the administration of anesthetics among surgeons and anesthesiologists, and that this likely influenced the timing of AP [21]. Incorporation of AP into the routine operating room workflow and administration of prophylaxis in the pre-operative area rather than in the operating room may ensure complete infusion of antibiotics prior to incision. As anesthesiologists play a critical role in postoperative infection control, the delegation of AP administration to the anesthesiology team should be considered. Nemeth et al. evaluated use of a verbal AP reminder in the surgical time-out process, but found that this intervention did not improve timeliness of administration of AP [21]. Nair et al. demonstrated the effectiveness of direct email feedback, antibiotic compliance reports, and real time alerts in improving antibiotic timing [22].

Our findings of variation in AP practices are similar to that of other studies evaluating the use of AP in pediatric surgical patients. Donà et al. noted variability in antibiotic prescribing for AP in their single-center study that evaluated the use of AP in children undergoing surgical procedures. The authors found that in the pre-intervention group, antibiotic selection was inappropriate in 51% of cases, and antibiotics were continued for a prolonged duration in 54.9% of cases [23]. Implementation of a clinical pathway proved to be a useful tool and led to a statistically significant improvement in the selection and duration of AP in pediatric patients; however, there still remained room for improvement of AP compliance in the post-intervention group [23]. Sandora et al. evaluated the national appropriateness of AP in children undergoing common surgical procedures using the Pediatric Health Information System database, and they noted significant variation in the use of AP across the 31 institutions submitting data [24]. AP was considered to be appropriate in only 64.6% of all cases in the study, with an inter-hospital variation ranging from 47.3% appropriateness to 84.4%. The authors noted that AP was commonly administered, even in cases for which AP was not indicated, revealing a significant overuse of antibiotics despite the presence of national guidelines and well described risks of antibiotic associated adverse reactions and secondary infections, such as *Clostridioides difficile* infection [24]. They also concluded that the lack of pediatric guidelines for AP may have impacted this finding of variability in AP practices between hospitals [24]. Additionally, while it is commonly inferred that the colonic composition in children is similar to that of adults, studies have demonstrated differences between the pediatric and adult gut microbiome [25,26]. Furthermore, the disproportionate differences in chronic conditions and comorbidities between children and adults may lend to different post-operative SSI risks when comparing these two populations [24]. Considering these differences, surgeons may be less inclined to extrapolate the adult guidelines to their pediatric patients.

AP was effective in the prevention of SSI in our study and only 6% developed an SSI. Of those children that developed an SSI, 80% were due to infections that were not covered by standard AP, 25% were premature infants, and 17% were in obese patients. It is well known that antibiotic overuse is frequent in neonates and significant variability exists in their use. Neonates are, therefore, at risk for antibiotic resistant organisms. Currently, there are limited data on appropriate surgical AP specific to neonates and AP in this population are based on adult guidelines [27]. Considering the unique microbiome of neonates and the morbidity associated with SSI in neonates, larger studies are warranted to determine effective AP in this particular population, as conventional AP may not be optimal. Two of the seven obese patients in this cohort developed an SSI. Patients who are obese commonly undergo longer operative times and are at risk for increased complications and prolonged hospitalizations following surgery [28]. Furthermore, the lack of data regarding antibiotic dose adjustments in obesity lends to the concern that these patients may not have adequate serum drug concentrations when standard doses of AP are utilized. Based on our small study, these special populations may benefit from a more tailored AP regimen.

This study has several limitations. This was a single-center study and, hence, the findings may not be generalizable to all pediatric surgical settings. Due to the retrospective study design, we were limited to information reported in the patients’ medical records; therefore, findings may have been misclassified if the data points were not completely recorded in the chart. The application of a clinical chart review may not have captured all facets of SSI documentation. We did not evaluate the use of oral antibiotics for mechanical bowel prophylaxis prior to elective colorectal procedures, so it is unclear if those practices were impactful in preventing SSI in our cohort. Finally, SSI cases were identified using a list provided by our infection preventionists using NHSN criteria; however, cases may not have been captured if cultures were not obtained despite objective signs leading to clinical suspicion of infection, such as fever or wound drainage.

## 5. Conclusions

In this study, we have identified multiple areas for improvement regarding the administration of AP in children undergoing colorectal surgeries. Lack of compliance with national guidelines for AP in children undergoing colorectal surgeries was high. A multidisciplinary approach to the development of standardized protocols, educational interventions, and EHR-based algorithms may facilitate or improve appropriate AP use. Special populations, such as neonates and obese children, may benefit from a tailored regimen for AP, as these children may be at risk for SSI due to organisms not covered by conventional AP regimens. Our findings indicate the need for larger studies to investigate optimal AP choices in special populations and to determine interventions to improve the provision of AP in children.

## Figures and Tables

**Table 1:** Demographic and clinical characteristics of study cohort.

Variable	Patient (*N* = 192)	Patient (%)	SSI (*N* = 12)	SSI (%)	*p*-value
**Age (range)**	4.7 mo (0–17.7 yr)		3.0 yr (1.8 mo−17.1 yr)		0.009
**Male**	113	59	9	75	0.365
**Race**					0.0592
**White**	128	67	5	42	0.058
**Black**	37	19	4	34	0.251
**Hispanic**	18	9	2	17	0.312
**Asian**	2	1	1	8	0.121
**Other**	7	4	0	0	1.000
**Co-morbidities/Exposures**					
**Proton Pump Inhibitor**	37	19	3	25	0.704
**Hyperglycemia**	37	19	5	42	0.042
**Immunocompromised**	14	7	1	8	1.000
**Steroids**	19	10	2	17	0.337
**Prematurity**	90	47	3	25	0.143
**Obese (BMI >30)**	7	4	2	17	0.063
**Beta-Lactam Allergy Reported**	10	5	1	8	0.484
**Previous hospitalizations within the year**	62	32	5	42	0.473
**Median Duration of Surgical Hospitalization**	13 (1–511) days		19 (3–92) days		0.526
**Median Surgery Duration (range)**	92 (20–579) min		112.5 (76–206) min		0.037
**Urgency**					<0.001
**Elective**	175	91	7	58	<0.001
**Emergent**	17	9	5	42	<0.001
**Surgery Type**					
**Appendectomy**	11	6	0	0	1.000
**Small Bowel Resection**	33	17	5	42	0.020
**Colon/Rectal resection**	84	44	8	67	0.134
**Chait Cecostomy**	7	4	0	0	1.000
**Soave**	5	3	0	0	1.000
**Duodenal atresia repair**	16	8	0	0	0.604
**Ostomy formation/revision**	67	35	4	33	1.000
**Ostomy closure**	66	34	3	25	0.550
**Exploratory laparotomy**	54	28	8	67	0.005
**Gastrostomy tube placement/Revision**	14	7	1	8	1.000
**Other**	43	22	3	25	0.733
**Wound Classification**					0.0005
**Clean-contaminated**	130	68	3	25	0.002
**Contaminated**	33	17	2	17	1.000
**Dirty**	29	15	7	58	0.000016
**ASA Class**					0.549
**I**	8	4	1	8	0.409
**II**	80	42	5	42	1.000
**III**	84	44	6	50	0.652
**IV**	20	10	0	0	0.618

**Table 2:** Appropriateness of antibiotic prophylaxis in children undergoing colorectal surgery.

	Appropriate	Inappropriate	SSI Appropriate	SSI Inappropriate
	*N* = 192 (%)	*N* = 192 (%)	*N* = 12 (%)	*N* = 12(%)
Antibiotic Selection	69 (36)	123 (64)	8 (67)	4 (33)
Antibiotic Dose	124 (65)	68 (35)	6 (50)	6 (50)
Antibiotic Timing	85 (44)	107 (56)	7 (58)	5 (42)
Antibiotic Duration	122 (64)	70 (36)	10 (83)	2 (17)

Note: For dual combinations, both antibiotics had to be appropriate.
